# Identification of curaxin as a potential new therapeutic for JAK2 V617F mutant patients

**DOI:** 10.1371/journal.pone.0286412

**Published:** 2023-05-30

**Authors:** Stella Pearson, Rognvald Blance, Fei Yan, Ya-Ching Hsieh, Bethany Geary, Fabio M. R. Amaral, Tim C. P. Somervaille, Kristina Kirschner, Anthony D. Whetton, Andrew Pierce

**Affiliations:** 1 Stem Cell and Leukaemia Proteomics Laboratory, Faculty of Biology, Medicine and Health, University of Manchester, Manchester, United Kingdom; 2 School of Cancer Sciences, University of Glasgow, Glasgow, United Kingdom; 3 Cancer Research UK Beatson Institute, Glasgow, United Kingdom; 4 Stoller Biomarker Discovery Centre, University of Manchester, Manchester, United Kingdom; 5 Leukaemia Biology Laboratory, Cancer Research UK Manchester Institute, Manchester, United Kingdom; Stanford University, UNITED STATES

## Abstract

Myelofibrosis is a myeloproliferative neoplasm (MPN) which typically results in reduced length and quality of life due to systemic symptoms and blood count changes arising from fibrotic changes in the bone marrow. While the JAK2 inhibitor ruxolitinib provides some clinical benefit, there remains a substantial unmet need for novel targeted therapies to better modify the disease process or eradicate the cells at the heart of myelofibrosis pathology. Repurposing drugs bypasses many of the hurdles present in drug development, such as toxicity and pharmacodynamic profiling. To this end we undertook a re-analysis of our pre-existing proteomic data sets to identify perturbed biochemical pathways and their associated drugs/inhibitors to potentially target the cells driving myelofibrosis. This approach identified CBL0137 as a candidate for targeting *Jak2* mutation-driven malignancies. CBL0137 is a drug derived from curaxin targeting the Facilitates Chromatin Transcription (FACT) complex. It is reported to trap the FACT complex on chromatin thereby activating p53 and inhibiting NF-kB activity. We therefore assessed the activity of CBL0137 in primary patient samples and murine models of *Jak2*-mutated MPN and found it preferentially targets CD34+ stem and progenitor cells from myelofibrosis patients by comparison with healthy control cells. Further we investigate its mechanism of action in primary haemopoietic progenitor cells and demonstrate its ability to reduce splenomegaly and reticulocyte number in a transgenic murine model of myeloproliferative neoplasms.

## Introduction

Myelofibrosis (MF) is a clonal stem cell associated myeloproliferative neoplasm (MPN) characterised by increased production of myeloid cells which leads to clinical features such as splenomegaly and bone marrow fibrosis [1]. MF is typically associated with expression of driver oncogenes including *MPLW515L* [2] and *Jak2* mutations [3]. JAK2 is a protein tyrosine kinase and MPL is the receptor for thrombopoietin which activates pleiotropic signalling events, including JAK2 signalling. Approximately 50% of patients presenting with MF have an activating JAK2V617F mutation [3–5]. The introduction of JAK2 inhibitors has been of benefit in the treatment of MF patients but they do not provide a cure. In fact, all current treatment strategies for MPN manage the disease but fail to affect a cure as they do not extinguish the myeloproliferative clone and unfortunately are ineffective in preventing the transformation to more acute diseases. There is therefore still an unmet need for additional targeted therapies and combination treatments to suppress or eradicate the cells at the heart of MF.

CBL0137, a metabolically stable curaxin, has been shown to have antitumour activity and is in phase I clinical trials in solid tumours (Clinical Trial Identifier: NCT01905228) and haematological malignancies, including AML and CLL (Clinical Trial Identifier: NCT02931110). CBL0137 has pleiotropic effects mediated via inhibition of the Facilitates Chromatin Transcription complex (FACT) [6]. These effects include the activation of TP53 and the inhibition of NF-ĸB activity and MYC expression [6–8]. FACT is a chromatin remodeling complex consisting of two subunits, SSRP1 and SPT16, whose expression is low in most adult tissues but has been shown to be up-regulated in many cancers and to be highly expressed in primitive cells [7, 9]. We have previously shown the potential of targeting TP53 and MYC to extinguish the leukaemic clone in a novel drug combination in other MPNs [10, 11]. Also, a recent study proposed that targeting TP53 in combination with the TNF-related apoptosis inducing pathway could potentially be an effective strategy for treating MF patients [12]. Furthermore, SSRP1 a subunit of the FACT complex, was identified as one of the top 20 hits from an shRNA screen to identify critical genes in myelofibrosis [13] and since NF-ĸB has been reported to be activated in MF [14, 15] we hypothesised that CBL0137 may have utility in the treatment of MF. A major advantage of this study is that CBL0137 is in clinical trial in other malignancies and displays limited toxicity in phase I clinical trials [16]. There is therefore potential to repurpose this drug, bypassing many of the hurdles present in normal drug development.

## Material and methods

### Patient material and primary cell assays

Use of human tissue was in compliance with the ethical and legal framework of the Human Tissue Act and the authors had no access to information that could identify individual participants. Experiments had ethical approval from the NRES committee of the regional NHS health research authority (14/LO/0489 and 17/LO/0888). Primary JAK2V617F positive samples were obtained from the Manchester Cancer Research Centre Biobank (HTA 30004) following authorisation by the Tissue Biobank’s scientific sub-committee. Samples were collected between July/2014 and May/2019 and Patient details are outlined in S1 Table in S1 File. The CD34+ cell population was enriched using CliniMACS (Miltenyi Biotec) according to standard protocols. Control samples were either surplus cells isolated from leucocyte cones from patients undergoing leukapheresis within the NHS Blood and Transplant Service or peripheral blood mobilised CD34+ cells surplus to requirements from patients undergoing chemotherapy and autologous transplantation for lymphoma. Written informed consent was obtained for all samples. Colony forming assays and cell tracer experiments were performed as previously described [11] and in the Supplementary Information. In brief colony forming assays were performed in methylcellulose complete media (R&D systems) supplemented with 2u/ml EPO at a density of 3000cells/ml. Cell proliferation and differentiation over 8 days in liquid culture were assessed using a CellTrace Violet Cell Proliferation Kit (Molecular Probes) and CD34-APC (eBioscience).

### Protein measurement

Protein expression was assessed either using capillary-based technology of Peggy-Sue (Protein Simple) or flow cytometry with the LSRFortessa^TM^ (Becton Dickenson) using standard protocols as previously described [11, 17] and in the Supplementary Information. Antibodies used are outlined in S2 Table in S1 File.

### p53 reporter assay

p53 transcriptional activity was assessed using Ba/F3 *JAK2V617F* cells stably expressing the pGreenfire1^TM^ p53 reporter construct (Systems Biosciences) using the Luciferase Assay System (Promega). Details are in the Supplementary Information. In brief, cells were harvested during log phase growth and 6x10^5^ cells (at 2x10^5^/ml) treated with 5μM Nutlin or 100nM CBL0137 for 6 hours prior to analysis of reporter activity using the Luciferase Assay System (Promega) as per manufacturer’s instructions. Luminescence was measured on a FLUOstar Omega plate reader (BMG Labtech) using a 10-second measurement read for luciferase activity.

### SWATH mass spectrometry

Mass spectrometry analysis was undertaken on HL-60, Mo7e and KG-1a cells treated with 100nM CBL0137 for 18 hours. Analysis was performed by SWATH MS using a TripleTOF 6600 mass spectrometer (Sciex) coupled on-line to an Ultimate 3000 HPLC (Dionex) as described in S1 File. Spectral data files were analysed using openSWATH (version 2.0.0) using the pan human library [18] followed by scoring and filtering using pyProphet (version 0.18.3). Protein abundance summarisation and normalisation were performed in R (version 3.4.1) using the packages SWATH2stats and MSstats from Bioconductor (version 3.5). Data was filtered using the SWATH2stats function ’filter-mscore_fdr’ using an overall protein FDR of 2% and an upper overall peptide FDR of 5%. Proteomics data have been deposited to the ProteomeXchange Consortium (http://proteomecentral.proteomexchange.org) via the PRIDE partner repository with the identifier PXD027250.

### RNA measurements

RNA analysis was undertaken on total RNA extracted from CD34+ cells isolated from peripheral blood using Qiagen RNeasy Plus kit. RNA sequence data were generated by the Genomic Technologies Core Facility at The University of Manchester as described in Supplementary Information. Raw counts were normalised and differential analysis undertaken in DEseq. Genes with a basemean above the 1st quantile were considered differentially expressed when displaying a fold change greater than 2 with an adjusted p-value lower than 0.01. The RNA sequencing data have been deposited at ArrayExpress (https://www.ebi.ac.uk/arrayexpress/) with the identifier E-MTAB-11653.

### *In vivo* studies

All mouse experiments were performed under a UK Home office project licence (licence PD6C67A47; protocols 2 and 5) in accordance with the UK Home Office regulations and ARRIVE guidelines. All experiments were subject to review by the with Animal Welfare and Ethical Review Board (AWERB) of the University of Glasgow. Mice were housed in conventional cages within a licenced, pathogen-free facility, under a 12hr light-dark cycle, at stable temperature (19–23°C) and humidity (55±10%) with ad libitum access to food and water. Numerous murine JAK2V617F knock-in models exist that demonstrate that heterozygous *Jak2V617F* mutation is sufficient to produce a myeloproliferative like disease but that acquisition of *Jak2V617F* homozygosity results in a switch from an Essential Thrombocythemia-like to Polycythemia Vera-like phenotype with the development of a profound and transplantable erythrocytosis. We therefore used homozygous mutant mice available via the *Jak2*^mut/+^Stella^Cre/+^ mouse model as previously reported [19]. Mice were bred on a mixed C57B16 background and experiments performed on 8–10 week old *Jak2V617F* homozygous mice [22]. Mice were monitored daily scoring for distress and weight was recorded weekly to ensure the animals were as healthy as possible and to alleviate unnecessary suffering. A humane endpoint was established as 10% loss of body weight or a health score of ≥10. This was not reached at any point during the study. CBL0137 or vehicle was administered once weekly for four weeks via intravenous injection at 40mg/kg for the first week, followed by doses of 50mg/kg in weeks two to four.

Mice were euthanised by CO_2_ inhalation in a CO_2_ chamber, cervically dislocated and then weighed. Blood was collected immediately via cardiac puncture for whole blood analysis (EDTA coated tubes; Sarstedt). Blood was diluted with 2mM EDTA and 100ul transferred to coated capillary tubes and run on the Procyte Dx Hematology Analyzer (IDEXX) using standard protocols.

## Results

### CBL0137 has differential effects on cells from MF patients

**Fig 1:**
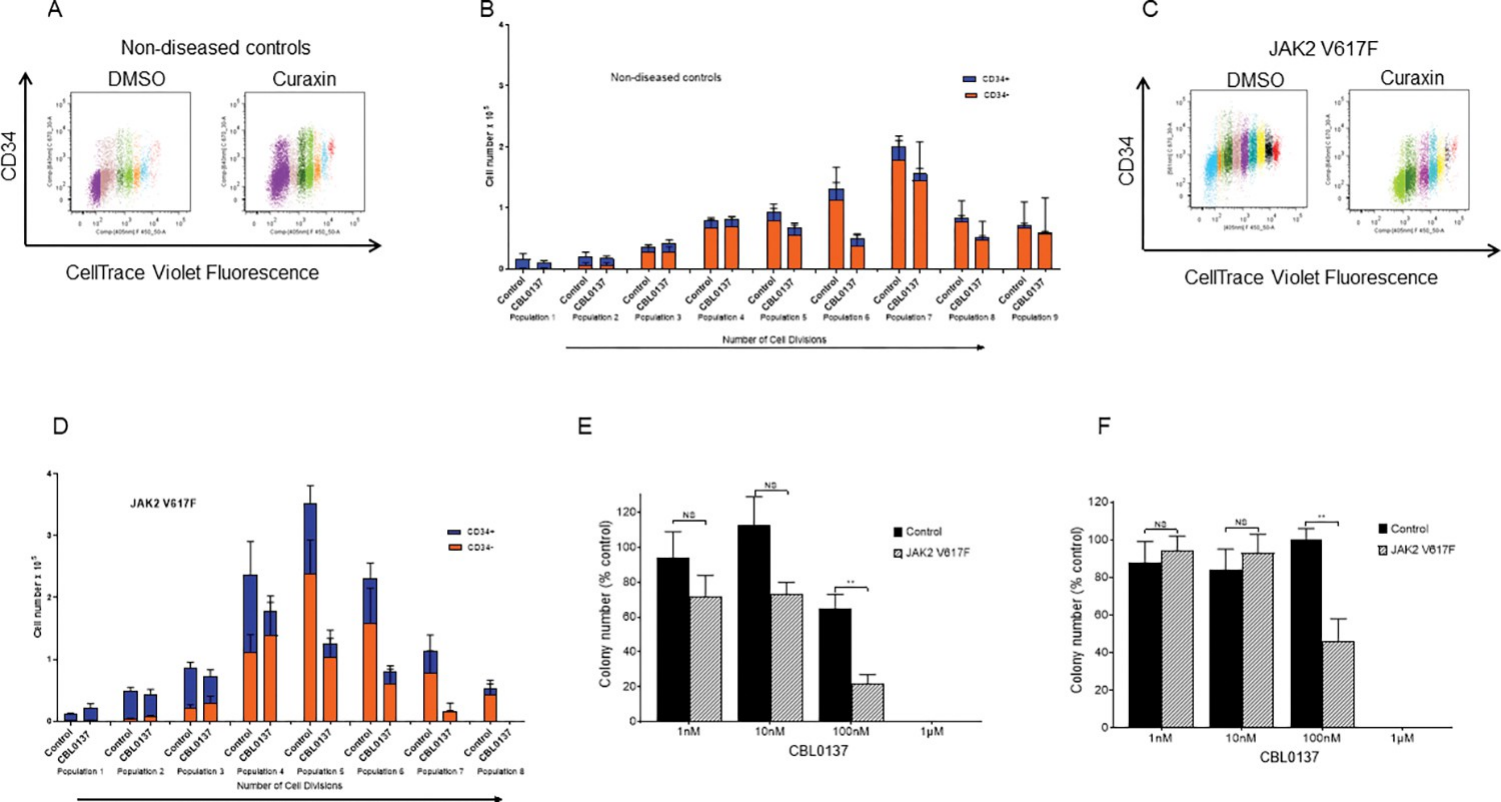
CBL0137 preferentially inhibits the colony forming ability of CD34+ cells isolated from MF patients. The effect of inhibition of the FACT complex (100nM CBL0137) on the cell division and differentiation of CD34+ cells isolated from healthy controls (Fig 1A and 1B) and JAK2V617F expressing patients (Fig 1C and 1D) was assessed in liquid culture. CD34+ cells were stained using CellTrace then seeded at a density of 1.5x10^5^/ml in IMDM, 20% FCS, rhIL-3 (20ng.ml), rhSCF (50ng/ml) and Flt-3 ligand (10ng/ml). Effects on cell division and CD34 expression were measured by flow cytometry at 8 days. Representative flow cytometric analysis (Fig 1A, 1C) of CD34 expression in relation to cell division (each colour representing a cell division) and the amalgamated results of the effects on cell division and CD34 expression (Fig 1B, 1D) are shown. Amalgamated results are shown as Mean±SEM (n = 4 for healthy controls and n = 7 for *JAK2V617F* mutated MF patients). The ability of CD34+ cells from *JAK2V617F* positive patients and non-leukaemic patients to form colonies in methylcellulose was assessed in the presence of a range of doses of CBL0137. CD34^+^ cells were plated in methylcellulose complete media (R&D systems) supplemented with 2u/ml EPO at a density of 3000cells/ml. Colonies were counted after 7 (Fig 1E) and 21 (Fig 1F) days and the data is displayed as the total number of colonies expressed as a percentage of the carrier control (mean±SEM, n = 4). The average number of colonies in the carrier control at day 21 was 69±16 for the *JAK2V617F* mutated MF patient samples and 31±9 for the healthy controls and (mean±SEM, n = 4). T-test results are represented by **<0.01.

To investigate the effects of CBL0137 in MF and attempt to define its mechanism of action initial experiments were undertaken in liquid culture. CD34+ cells isolated from peripheral blood of JAK2V617F mutated myelofibrosis patients and non-diseased controls were labelled with CellTrace^TM^ to allow analysis of both cell division and differentiation (Fig 1A–1D). As we have shown previously when investigating the effects of CBL0137 in other diseases [20] with different control samples CBL0137 has minimal effect on healthy controls (Fig 1A and 1B). In comparison, CBL0137 demonstrated antiproliferative activity in JAK2V617F-mutated cells from MF patients, and in addition reduced the percentage of CD34+ expressing cells (Fig 1C and 1D).

Given these differential effects on the primitive CD34 expressing cells we next investigated the effects of CBL0137 on haemopoietic colony formation at a range of drug doses. As we have shown previously CBL0137 has a minimal effect on healthy control cells up to a dose of 100nM [20]. However, at 100nM CBL0137 significantly inhibits colony formation in the CD34+ cells isolated from the JAK2V617F expressing patients (Fig 1E and 1F).

These data demonstrate that, compared with normal CD34+ stem and progenitor cells, *Jak2*-mutated CD34+ cells from MF patients exhibit selective sensitivity to CBL0137 *in vitro*.

### CBL0137 mode of action in JAK2V617F expressing cells

**Fig 2:**
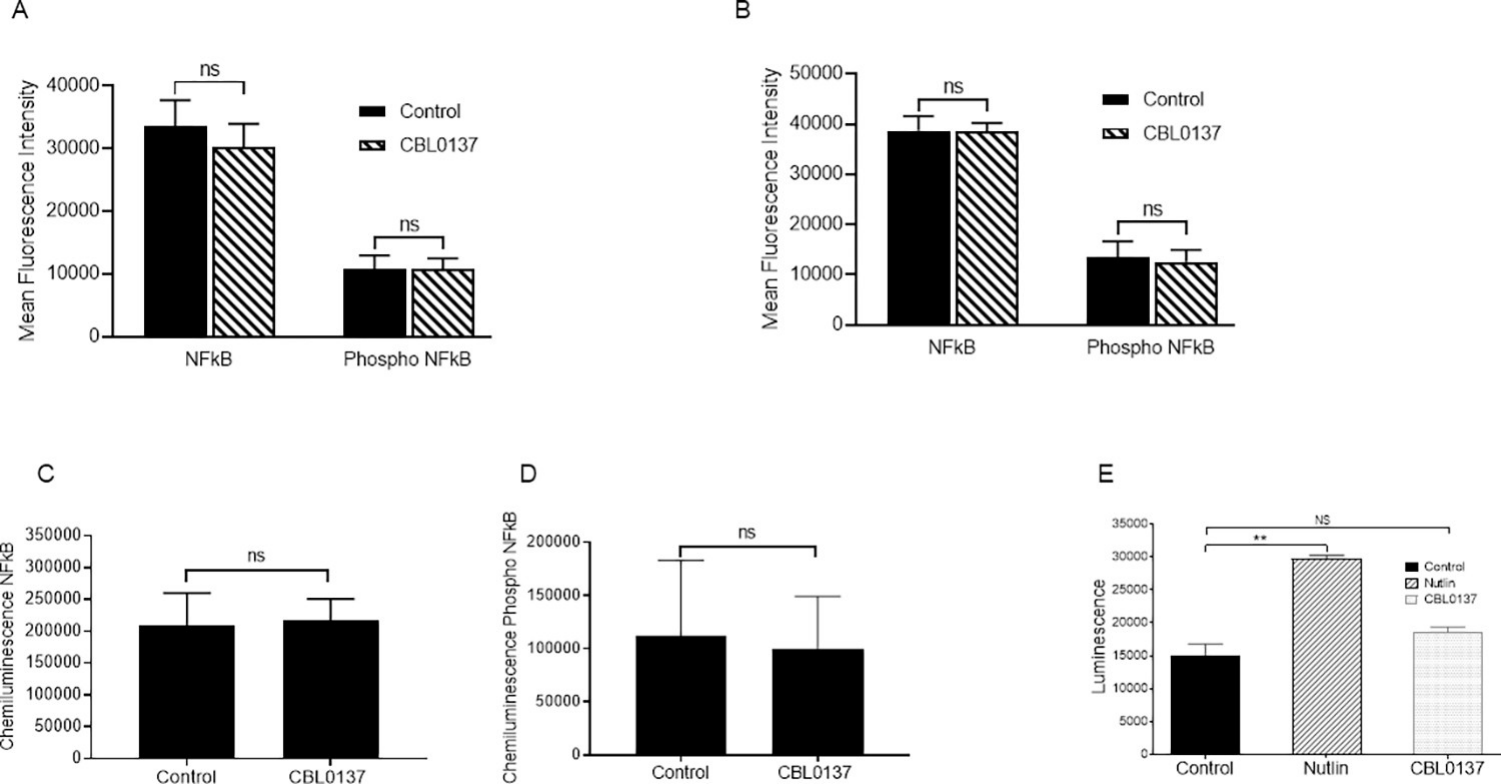
CBL0137 does not activate p53 or affect NF-ĸB expression in CD34+ cells isolated from MF patients. CD34+ cells isolated from *JAK2V617F* mutated MF patients were treated for 1, 6 and 24 hours with 100nM CBL0137 and the effects of drug treatment on NF-ĸB, and phospho S536 NF-ĸB expression analysed. The effects at 1 hour (Fig 2A) and 24 hours (Fig 2B) were assessed by flow cytometry and the results are displayed as mean fluorescence intensity (mean±SEM, n = 3). The effects of 6 hours of CBL0137 treatment on NF-ĸB (Fig 2C) and phospho S536 NF-ĸB (Fig 2D) were assayed using Peggy-Sue (Protein Simple). The expression levels were normalised using actin and are shown using the chemiluminescent peak area (mean±SEM, n = 4). Ba/F3 JAK2V617F cells expressing the p53 reporter pGreenFire1 p53-GF-EF1-Puro (Systems Biosciences) were treated with 100nM CBL0137 for 6 hours prior to analysis of reporter activity using the Luciferase Assay System (Promega) as per manufacturer’s instructions. Cells were treated with the p53 activator Nutlin (5μM for 6 hours) as a positive control. Results are shown as total luminescence (mean±SEM, n = 3). T-test results are represented by **<0.01.

Curaxins are reported to exert their antitumour activity in a cell specific context by the inhibition or activation of a wide range of targets (reviewed by Jin *et*.*al*. [21] and Oien *et*.*al*. [22]). Given the differential effects of CBL0137 between cells isolated from JAK2V617F expressing patients and heathy controls, we next investigated its mode of action in haemopoietic CD34+ progenitor cells. As CBL0137 has been widely reported to achieve its therapeutic effects by activating TP53 and inhibiting NF-ĸB in other systems, we investigated these two pathways. The effects of 1, 6 and 24 hour incubation with CBL0137 (100nM) on the expression of NF-ĸB-p65 and phospho NF-ĸB-p65 (a surrogate marker for activation/inhibition), were investigated by flow cytometry (Fig 2A and 2B) and capillary based electrophoresis immunoassay (Fig 2C and 2D). Fig 2A and 2B show the amalgamated data from three flow cytometry experiments with individual representative FACS plots being displayed in S1 Fig in S1 File. At 100nM, CBL0137 does not affect NF-ĸB-p65 expression or phosphorylation status at either one (Fig 2A), six (Fig 2C and 2D) or 24 hours (Fig 2B).

To investigate the potential effects of CBL0137 on TP53 activation we chose to use a luciferase TP53 reporter system in a JAK2V617F expressing cell line model we have previously successfully used to identify drug targets in MPN [11, 17, 23]. Cells were treated with CBL0137 (100nM) or Nutlin (5μM) as a positive control. Nutlin inhibits the interaction between MDM2 and TP53 leading to the stabilisation of TP53 [24]. Whilst Nutlin leads to an increase in TP53 transcriptional activity CBL0137 fails to induce TP53 transcriptional activation (Fig 2E). Although p53 activation was used to screen for curaxins, curaxin toxicity was not limited to cells expressing wild-type p53 or inhibited by over-expression of BCL2 suggesting p53-induced apoptosis does not contribute to curaxin’s effects [6]. To identify potential TP53 independent modes of action we undertook a proteomic screen of the effects of CBL0137 on TP53 null, mutant and wild type haemopoietic cell lines reasoning that any effects seen in all three cell lines would represent TP53 independent mechanisms.

### Proteomic analysis of CBL0137 mode of action in relation to TP53 activity

We undertook a proteomic screen by SWATH-based mass spectrometry on three myeloid cell lines of different TP53 mutational status (TP53 wild type, TP53 null and TP53 mutant) treated with and without CBL0137 for 18 hours. Mo7e are wild type for TP53, HL-60 TP53 null and KG-1a contain a 672+1G>A mutated TP53 (S2 Fig in S1 File). Following 18-hour treatment with 100nM CBL0137 no difference in cell viability was observed (Mo7e 94%, HL-60 97%, KG-1a 98%). These results are in line with data from Eriksson et al who reported similar IC_50s_ on wild type, mutant and p53 null AML cell lines with the curaxin quinacrine [25].

**Fig 3:**
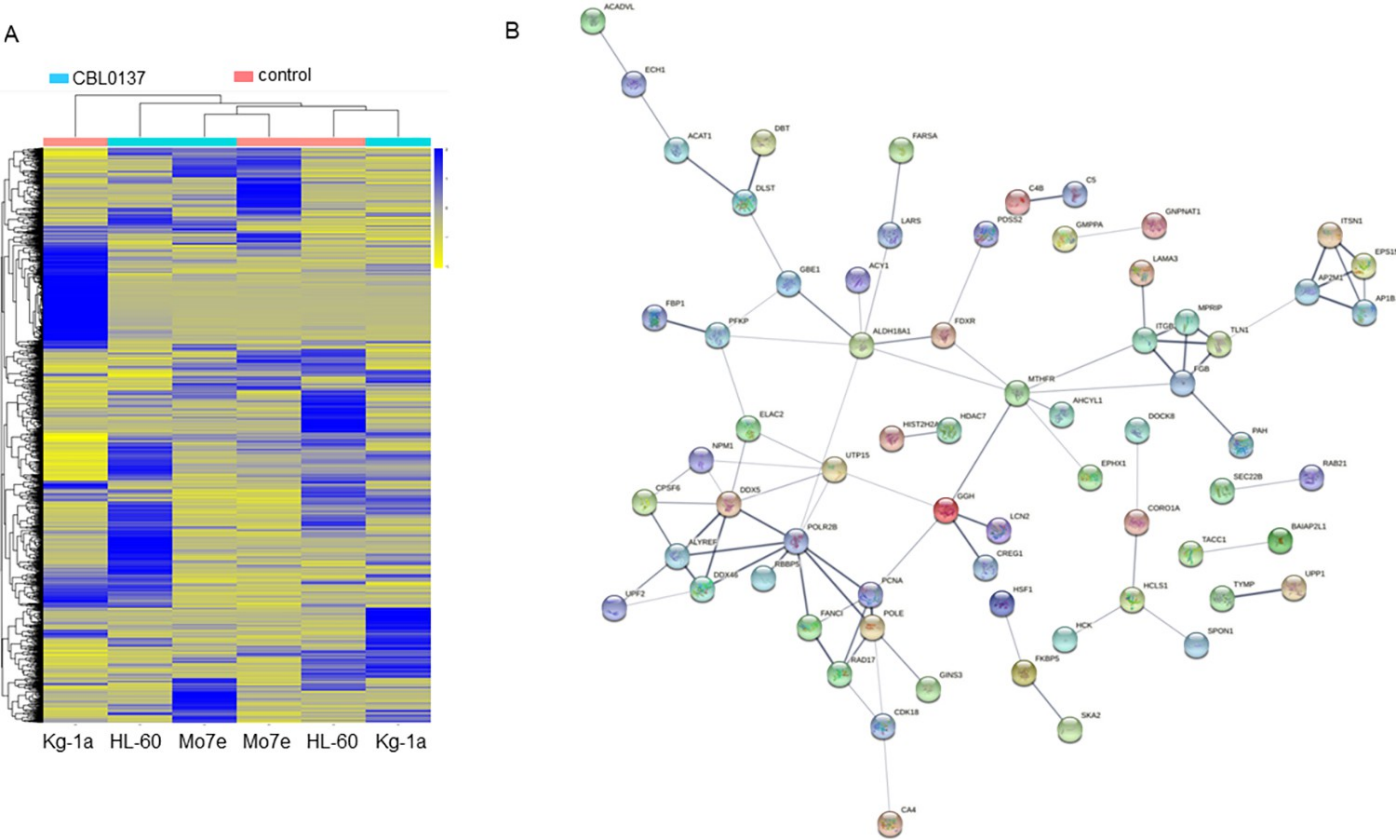
Proteomic data suggests a role for RNA processing in CBL0137 mode of action. Fig 3A; Nuclear and cytoplasmic enriched protein lysates from three cell lines HL-60, Mo7e and KG-1a treated with 100nM CBL0137 for 18 hours were subject to SWATH MS analysis. Results are displayed as a heatmap with individual samples as columns and scaled normalised relative protein abundance levels as rows. Columns are colour coded according to drug status of the sample. Protein abundances are coloured as blue for high abundance and yellow for low abundance. Clustering of columns and rows were performed using Manhattan distance. Rows were scaled by an initial log2 transformation before the subtraction of the mean then dividing by the standard deviation. Fig 3B; The 94 proteins defined as changing (2-fold) on CBL0137 treatment in all three cell lines were analysed in the protein interaction software ‘‘STRING” (https://string-db.org). Active interaction was set at ‘‘medium” with the sources for the interaction restricted to text mining, experiments, databases, co-expression and neighbourhood. The thickness of the lines between proteins represents the confidence of the protein-protein association (0.4, 0.7, 0.9).

In total 7265 proteins were identified across all conditions (S3 Table in S1 File). Average intensity across all 12 samples was 16527+/-3089 (mean+/-SD, a CV of 19%). Counting proteins seen in at least one experimental condition, 6393 proteins were identified in the nuclear fraction and 3665 in the cytoplasmic fraction (seen in at least one sample in one cell line). Whilst there was considerable overlap in the proteins identified in the two cellular compartments Manhattan clustering of the results on expression intensity showed a clear separation on nuclear/cytoplasmic samples (S3 Fig in S1 File). When clustering was performed on all the proteins identified (Fig 3A) protein samples did not separate on cell line or drug treatment suggesting a p53 independent mechanism pertained. Applying a cut-off of two-fold change in protein expression, 1% of the cytoplasmic proteins and 2% of the nuclear proteins were identified as changing in all three cell lines upon CBL0137 treatment (S3 Table in S1 File). Analysis of the protein interactions of those defined as changing in all three cell lines (TP53-independent), using STRING software (https://string-db.org) [26] identified significantly more interactions than would be expected at random with a PPI enrichment p-value of 0.0013. This analysis revealed a cluster with two arms based around RNA polymerase II (Fig 3B). The first arm containing PCNA, FANCI, DDX5, NPM1, and RAD17 suggests the cells are undergoing replication stress and a DNA damage-type response upstream of p53. It should be noted that PCNA showed both an increase in the nucleus and reduction in the cytoplasm in all three cell lines (S4 Table in S1 File). However, whilst the cells do indeed display an increase in γH2AX expression, an early marker of DNA damage, upon CBL0137 treatment (S4 Fig in S1 File) this does not lead to a p53-induced cell cycle blockade (S5 Fig in S1 File) as may have been anticipated.

The second arm is based around RNA processing and ALYREF/THOC4 (Fig 3B) and includes NPM1, DDX5 and CPSF6, which are all up-regulated in the three AML cell lines upon CBL0137 treatment. THOC4 is a member of the THO complex that is involved in the coupling of transcription to the export of mRNA from the nucleus into the cytoplasm [27]. The FACT complex is involved in transcription elongation [28] and it has been reported that FACT is required for binding of mRNA to THOC4 [29]. Further we have previously shown disruption of RNA processing in MPLW515L oncogene-driven myeloproliferative disorders via a novel THO complex pathway [23] and that THOC5 is not only a common phosphorylation target of numerous leukaemogenic oncogenes [30] but also plays a role in *Myc* regulation [23, 31] a target of CBL0137 [32]. Moreover, we have recently shown that THOC5-DDX5 interaction (another protein changing here in all three cell lines on CBL0137 treatment) is essential for RNA polymerase II transcription elongation [33] which is a known target of the structurally related drug Quinacrine [25]. Therefore, to gain a deeper understanding of the potential role of CBL0137 in RNA processing we undertook an RNA sequencing analysis on *Jak2*-mutated CD34+ cells isolated from MF patients treated ex-vivo with CBL0137 for 24-hours.

### Transcriptomic analysis of CBL0137 mode of action

**Fig 4:**
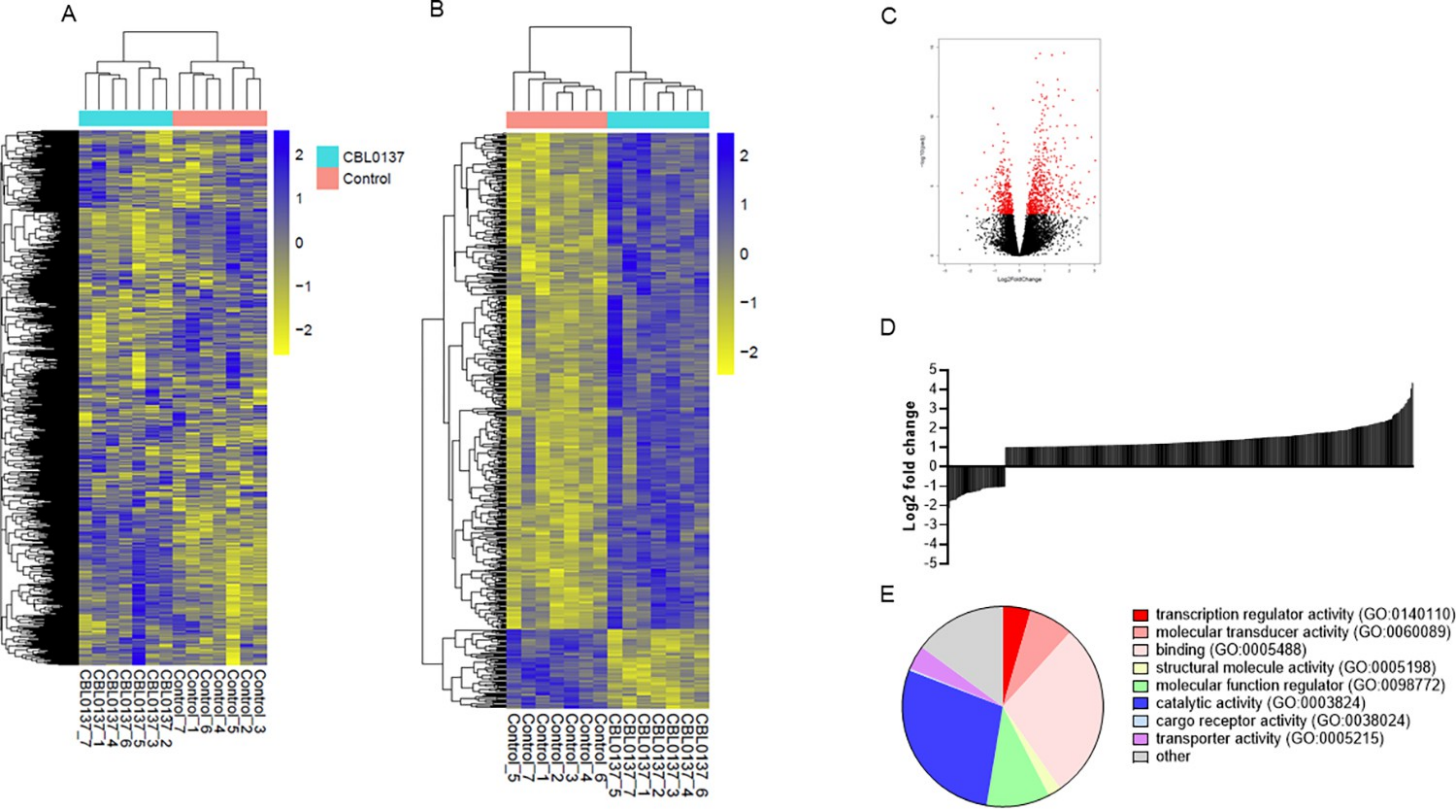
RNA sequence data analysis. CD34^+^ cells were enriched from the blood of JAK2V617F mutated MF patients and treated for 24 hours with 100nM CBL0137. RNA expression levels were measured using Illumina HiSeq NGS and differential analysis was undertaken in DESeq. Heat maps displaying the hierarchical clustering calculated using Euclidean distance measurements on RNA counts of all (22,088) the transcripts detected (Fig 4A) and the 551 transcripts defined as changing (2-fold adjusted p-value< 0.01). Annotations along the columns show patient and treatment number (Fig 4B). Fig 4C; Volcano plot of all identified transcripts plotting Log_2_ fold change on CBL0137 treatment against Log_10_ of the adjusted p value. Transcripts with an adjusted p-value<0.01 are shown in red. Fig 4D; Log2 distribution of transcripts displaying greater than a 2-fold change in expression with an adjusted p-value of <0.01. Fig 4E; Gene Ontology analysis of differentially expressed genes (2-fold adjusted p-value <0.01).

CD34+ cells were isolated from seven *Jak2*-mutated MF patients and treated with 100nM CBL0137 for 24 hours before extraction of RNA and analysis by Illumina RNA sequencing. Unsupervised analysis of the data showed a clear separation of carrier treated and CBL0137 treated patient samples (Fig 4A). Applying an FDR cut-off of <0.01 and defining a change as a 2-fold difference in expression, 551 transcripts were differentially expressed upon CBL0137 treatment (Fig 4B and S5 Table in S1 File). When applying only an FDR cut-off (padj <0.01) there was an approximately even distribution of down and up-regulated transcripts (1007 and 1289 respectively) as illustrated in Fig 4C. However, the majority of transcripts that displayed a 2-fold change with an adjusted p-value of less than 0.01 were increasing in CBL0137 treated cells (Fig 4D): 75 transcripts were down-regulated and 476 up-regulated. Notably, CBL0137 treatment led to down regulation of transcripts for FACT components SSRP1 and SUPT16H (1.8-fold decrease with a padj 2.4x10^-5^ and 1.3-fold with a padj of 1.2x10^-4^ respectively). Prominent amongst the majorly affected gene transcripts are several transcriptional regulators: *HIF3A* displays a 3.1-fold increase (padj 1.2x10^-12^) and *HEY1* a 4.9-fold decrease (padj 3.1x10^-5^). HIF3A is a negative regulator of HIF1A which has recently been suggested as a potential drug target in JAK2V617F driven MPN [34]. Furthermore, Baumeister et al [35] demonstrate that inhibition/or knockdown of *HIF1A* leads to cell cycle arrest in model systems and reduced clonogenic potential in cells from MPN patients. HEY1 is a downstream effector of Notch signalling which along with the Wnt pathway plays a critical role in haemopoietic differentiation. In fact, *DKK1*, an antagonist of the canonical Wnt signalling pathway, is also down regulated (3.2-fold decrease, padj 0.0098). Characterisation of the genes defined as changing highlights the appearance of transcription regulators (Fig 4E).

**Fig 5:**
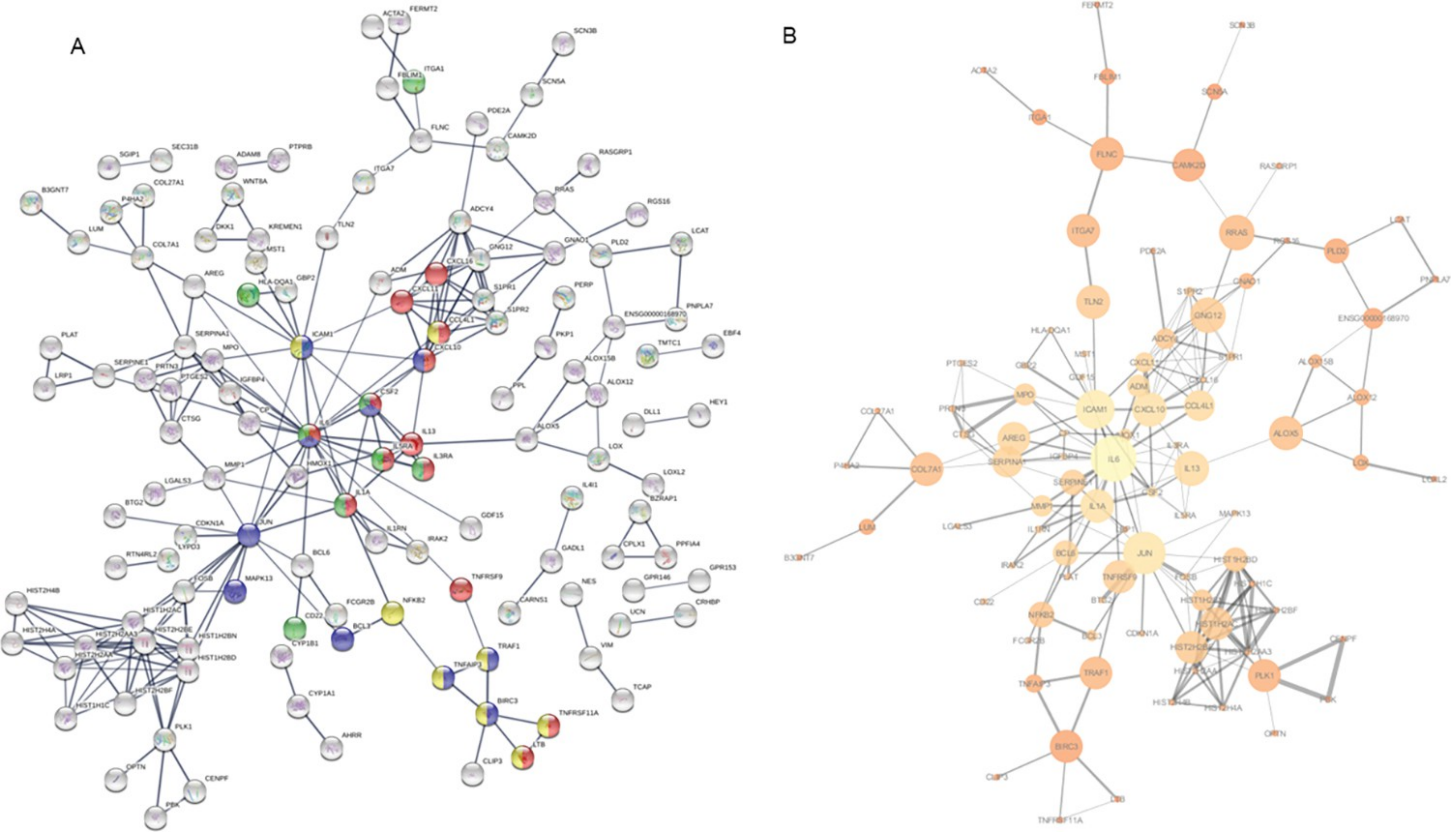
In silico analysis of RNAs identified as changing on CBL0137 treatment identifies a possible role for the non-canonical NF-kappa B signaling pathway. Fig 5A; The interactions between proteins corresponding to the genes identified as changing (2-fold with an FDR of 0.01) were identified using ‘‘STRING” (https://string-db.org). The thickness of the lines between proteins represents the confidence of the protein-protein association (0.4, 0.7, 0.9). The contribution of the nodes to the KEGG pathway enrichment analysis are illustrated by coloured nodes. Red–cytokine-cytokine receptor interaction (hsa04060), Blue–TNF signalling pathway (hsa04668), Yellow—NF-kappa B signaling pathway (has04064), Green Hematopoietic cell lineage (hsa04640). Fig 5B; The interacting proteins were exported to “Cytoscape” software to perform a network analysis. Nodes are sized and coloured according to their centrality values, the higher the value the larger the node and brighter the colour. The thickness of the edges illustrates the degree of co-expression.

To investigate functional aspects of gene expression changes STRING software [26] was employed to identify putative protein-protein interactions (Fig 5A). The major KEGG pathway identified was cytokine-cytokine receptor interaction (S6 Table in S1 File). Within the set of cytokines identified as changing the IL5/IL3/CSF2 pathway was perturbed at several levels. Degree network analysis of these interacting proteins identified IL6 and JUN with a high degree of connectivity. They had the highest betweenness and closeness centrality (Fig 5B) suggesting that they have a high extent of independence and are controlling the flow in the network. *Jun* is a direct target of NF-ĸB in haemopoietic cells [36] and is a negative regulator of HSC self-renewal. Whilst BIRC3, a protein that positively regulates the canonical NF-ĸB pathway and suppresses non-canonical NF-ĸB signalling, and NF-ĸB2 are present in the network they display low levels of betweeness and closeness centrality.

**Fig 6:**
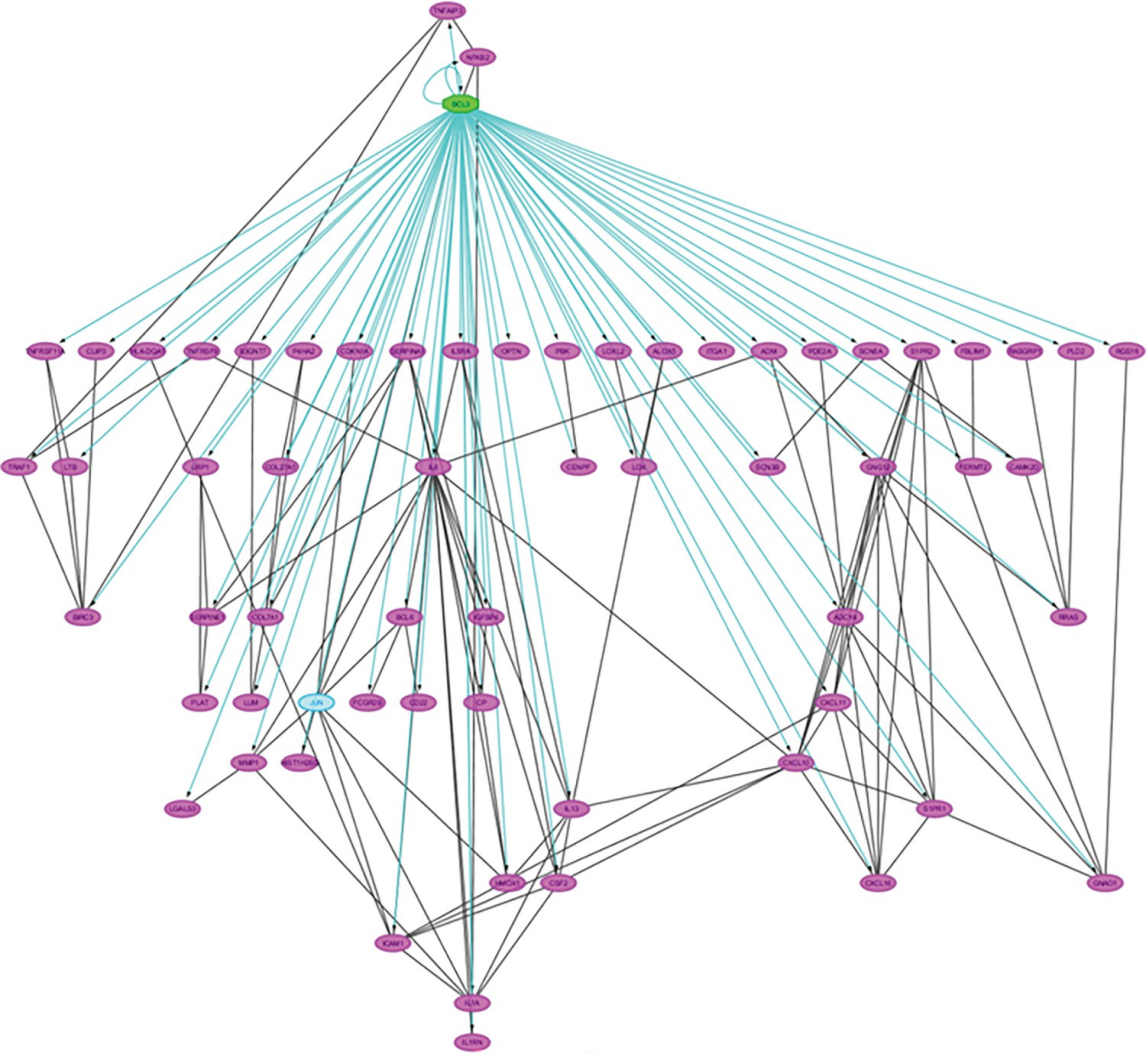
In silico analysis of the networks identified from the RNAs changing on CBL0137 treatment identifies BCL-3 as a possible regulator. The transcription factors predicted to regulate the genes within the network shown in Fig 5 were identified using the “iRegulon” application in Cytoscape and are displayed as a hierarchical network.

**Fig 7:**
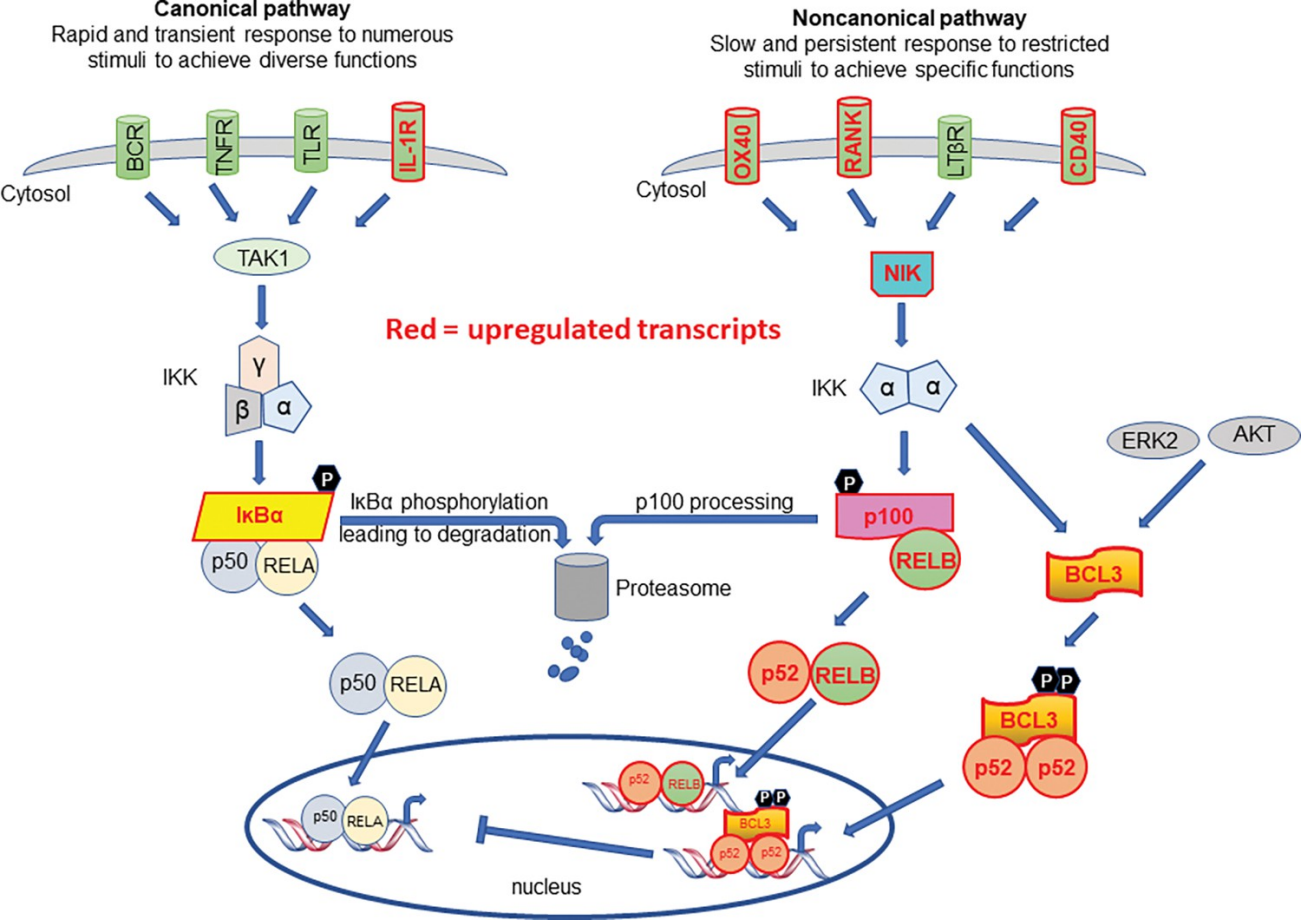
NF-ĸB signalling. Schematic representation of the canonical and non-canonical NF-ĸB signalling pathways with transcripts shown to be upregulated by CBL0137 highlighted in Red text and red outline.

To gain further insight into the mode of action of CBL0137 we undertook an *in-silico* analysis using Cytoscape to identify the transcription factors potentially involved in the observed changes. BCL3 was identified as the top scoring transcription factor. BCL3 contributes to the regulation of NF-ĸB target genes and in line with this NF-ĸB2 was also identified as a potential regulator of the induced changes (Fig 6). In addition to inhibiting the nuclear translocation of NF-ĸB1/p50 BCL3 also acts as a transcriptional regulator of NF-ĸB target genes in the nucleus. It should be noted that as well as being reported to be a potential master regulator of the changes observed, BCL3 itself is up-regulated 2.2-fold (padj 0.005) by CBL0137 treatment. The BCL3 transcriptional regulation of NF-ĸB target genes in the nucleus involves phosphorylation of BCL3 at ser446. Interaction of the phosphorylated BCL3 with NF-ĸB2 p52 homodimers results in the down regulation of many of the RelA/p50 target genes whilst leading to the activation of a subset of genes. Our data therefore appear to demonstrate that whilst there may be a repression of the canonical NF-ĸB pathway we are seeing an activation of the non-canonical pathway. Indeed, many of the components of the non-canonical pathway are up-regulated eg NF-ĸB2, RelB, and NIK (Fig 7 and S7 Table in S1 File).

Having defined some of the pleiotropic methods by which curaxins exert their cell specific antitumour activity in MF we initiated in vivo studies to further demonstrate the potential utility of CBL0137 as a novel treatment agent.

### Assessment of CBL0137 efficacy in an *in vivo* model of JAK2V617- driven malignancy

**Fig 8:**
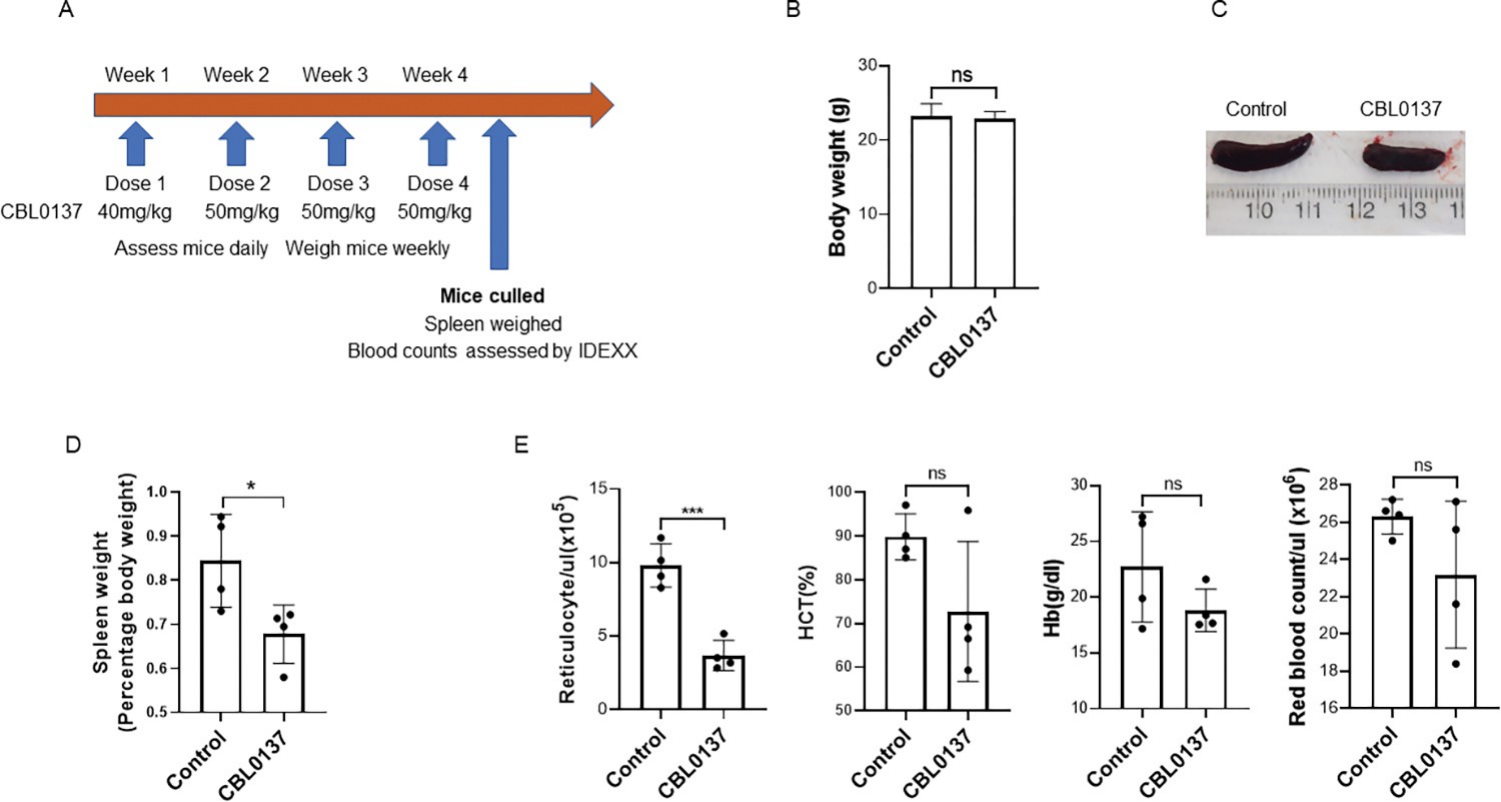
In vivo assessment of CBL0137. Fig 8A; Schematic representation of the treatment regime that 8–10-week-old mice homozygous for JAK2V617F were subjected to. Fig 8B; At the time of culling mice were weighed and the data is shown as mean weight+/-SEM (n = 4) for control and CBL0137 treated mice. Spleens were removed, photographed and weighed. Representative photographs of spleens (Fig 8C) and weights as a percentage of total body weight (Fig 8D) for control and CBL0137 treated mice are shown mean+/-SD (n = 4). Average spleen weights for vehicle treated mice were 198mg+/-19, and CBL0137 treated mice 155mg+/-8 (mean+/-SEM, n = 4). Fig 8E; Blood counts were analysed on a IDEXX ProCyte Dx analyser and are shown as mean+/-SD (n = 4).

To progress CBL0137 towards the clinic for the treatment of MF we used a preclinical mouse model of *Jak2V617F* driven MPN [37]. We adopted a published CBL0137 treatment regime (Fig 8A) from a preclinical testing program of *in vivo* solid tumours and acute lymphocytic leukaemia panels [38]. CBL0137 was well tolerated as indicated by daily monitoring of animal behaviour and exemplified by lack of weight loss during the treatment regime (Fig 8B). As previously published for this model system [19] mice homozygous for JAK2V617F displayed marked erythrocytosis with an average haematocrit of 89.8% ±2.6 (mean±SEM n = 4) and HB levels of 22.7g/dl ±2.4 (mean±SEM n = 4). Characteristic of MPN and this model the mice also display increased reticulocyte counts 9.8x10^5^/ml ±0.73 (mean±SEM n = 4) and splenomegaly 198mg±19 (mean±SEM n = 4) approximately twice the ‘‘normal” spleen weight. CBL0137 treatment significantly (p = 0.036) reduced the splenomegaly (Fig 8C and 8D) and reticulocyte (p = 0.0005) count (Fig 8E). Whilst not reaching statistical significance mice treated with CBL0137 also showed a downward trend in both haematocrit and Hb levels (Fig 8E). Thus, a four-week treatment of mice with CBL0137 was well tolerated and resulted in suppression of myeloproliferation, as evidenced by a reduction in spleen size and reticulocyte count.

## Discussion

Whilst genomic and transcriptomic screening has led to advances in our knowledge of leukaemic stem cell persistence and clonal evolution these finding have sometimes been disappointing in terms of drug target identification. We have successfully undertaken systematic proteomic screens in MPNs identifying novel drug targets [11, 23]. Ultimately these systematic discovery approaches represent an opportunity to identify and potentially repurpose drugs already in clinical trials for other diseases, thereby fast-tracking with respect to drug safety, pharmacokinetics and clinical activity. Here our aim was to assess the potential of CBL0137 as a new treatment strategy for JAK2V617F-mutated MF and define its mechanistic action in primary haemopoietic progenitors. Using primary patient material we were able to demonstrate that CBL0137 preferentially targets cells from *JAK2V617F*-mutated MF patients compared to healthy control cells. It should be noted that without the allele burden data for these patients we are able to report an MPN specific effect but unable to formally conclude this is a JAK2-specific effect. This data is unavailable for our patient cohort as allele burden was not routinely measured in the clinic in the UK at the time of sample collection.

Development of drug resistance and transformation to a more fatal disease are common problems encountered in precision medicine as applied to cancer treatment and highlight the need for understanding mechanisms of action for drugs employed in such strategies. It has been widely reported that curaxins and quinacrine, upon which they are based, exert their effects via the activation of TP53 and the inhibition of NF-ĸB activity. However, their effects appear context specific and are based on a plethora of targets including ribosomal biogenesis, autophagy and Hedgehog signalling [21, 22]. Our data indicates that CBL0137 does not exert it effects via TP53 in haemopoietic cell lines: curaxin toxicity was not limited to cells expressing wild-type p53 [6]. Further the antitumour activity of curaxins are not inhibited by overexpression of BCL-2 suggesting p53-induced apoptosis does not contribute to curaxins effects [6].

Whilst our data does not implicate TP53 in the CBL0137 mode of action, our proteomic analysis did suggest that the cells undergo replication stress and a DNA damage response upstream of TP53. Whilst curaxins intercalate into DNA and target FACT it has been reported they do not cause DNA damage [8]. However, this may be cell type specific as studies in breast cancer [39] and glioblastoma [40] report increases in DNA damage upon curaxin treatment. Also, transcriptomic analysis in medulloblastoma [13] demonstrated that CBL0137 suppressed DNA repair-related processes. Interestingly in this study they also showed cell type specific cell cycle effects with either a G1 or S/G2-M blockade depending on the cell line used.

Whilst our data also suggests that CBL0137 does not exert it effects via the canonical NF-ĸB pathway in haemopoietic cells it does infer that CBL0137 disrupts non-canonical NF-ĸB signalling. The canonical pathway represents a quick-fire response system whilst the non-canonical pathway is slow and persistent. Dysregulation of the non-canonical pathway contributes to inflammatory diseases [41]. In T cells the non-canonical NF-ĸB pathway has been shown to regulate CSF2 expression which is increased 4.2-fold (padj 0.00004) upon CBL0137 treatment in our experiments. Our transcriptomic data led to the identification of 7 of the 9 TNF superfamily members reported to induce the non-canonical pathway. Six of these receptors report a CBL0137 induced change in expression (5 up and 1 down). It has also been reported that increases of NIK expression (upregulated 1.5-fold padj 9.1x10^-6^ in our study) in AML activate the non-canonical pathway and supresses the canonical pathway [42, 43] which indicates that the canonical and non-cononical pathways have opposing effects in AML. It has been reported that Curaxins inhibit DNA methylation by DNMT3A [34] and that stabilisation of NIK effects *DNMT3A* expression [43]. Our data therefore infer that dysregulation of the non-canonical NF-ĸB pathway is a constituent of the action of CBL0137. Of direct relevance, modulating the non-canonical pathway in AML models does indeed supress the leukaemic phenotype [43].

To demonstrate in vivo effects of the drug we undertook studies in a preclinical mouse model of JAK2V617F driven MPN [19]. These experiments demonstrate that CBL0137 is well tolerated and was able to significantly reduce splenomegaly and the elevated reticulocytes observed in this model.

In conclusion our study illustrates the potential of CBL0137 as a possible therapeutic agent for the treatment of MPN and provides evidence to support the initiation of further studies on the use of curaxin in MPN treatment. Our current work is aimed at expanding these observations into other preclinical murine models of MPN.

## Supporting information

S1 Checklist The ARRIVE guidelines 2.0: Author checklist. (DOCX)

S1 File (RAR)

S1 Raw images Original uncropped, unadjusted images from the western blots shown in S2 and S4 Figs in S1 File. (PDF)
