# Supplementary material for: Identification of curaxin as a potential new therapeutic for JAK2 V617F mutant patients
Source: PLoS One. 2023 May 30;18(5):e0286412. doi: 10.1371/journal.pone.0286412 (PMC10228771; doi:10.1371/journal.pone.0286412)
Supplement: S1 Checklist — (DOCX) [file pone.0286412.s001.docx]

NOTE: Please save this file locally before filling in the table, DO NOT work on the file within your internet browser as changes will not be saved. Adobe Acrobat Reader (available free [here](https://acrobat.adobe.com/uk/en/acrobat/pdf-reader.html)) is recommended for completion.


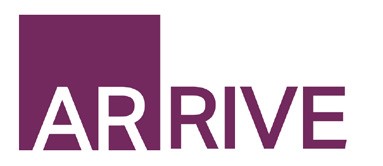
 The ARRIVE guidelines 2.0: author checklist

| The ARRIVE Essential 10 |
| --- |
| These items are the basic minimum to include in a manuscript. Without this information, readers and reviewers cannot assess the reliability of the findings. |

**Section/line**

**Item Recommendation number, or reason**

**for not reporting**

| **Study design** | 1 | a. | For each experiment, provide brief details of study design including:  The groups being compared, including control groups. If no control group has been used, the rationale should be stated. | a. C57/BL6 female Jak2V617F homozygous with and without curaxin treatment for 4 weeks.  b. Animals from several litters randomly grouped |
| --- | --- | --- | --- | --- |
|  |  | b. | The experimental unit (e.g. a single animal, litter, or cage of animals). |  |
| **Sample size** | 2 | a. | Specify the exact number of experimental units allocated to each group, and the total number in each experiment. Also indicate the total number of animals used. | 1. N=4 for each group |
|  |  | b. | Explain how the sample size was decided. Provide details of any *a priori* sample size calculation, if done. | 1. Estimated based on previous experiments and published data |
| **Inclusion and exclusion criteria** | 3 | a. | Describe any criteria used for including and excluding animals (or experimental units) during the experiment, and data points during the analysis. Specify if these criteria were established *a priori.* If no criteria were set, state this explicitly. | 1. Not relevant 2. Not relevant 3. N=4 |
|  |  | b. | For each experimental group, report any animals, experimental units or data points not included in the analysis and explain why. If there were no exclusions, state so. |  |
|  |  | c. | For each analysis, report the exact value of *n* in each experimental group. |  |
| **Randomisation** | 4 | a. | State whether randomisation was used to allocate experimental units to control and treatment groups. If done, provide the method used to generate the randomisation sequence. | 1. We didn’t use a randomization method. Random animals were assigned drug or vehicle. 2. Confounders were not controlled. |
| A a |  | b. | Describe the strategy used to minimise potential confounders such as the order of treatments and measurements, or animal/cage location. If confounders were not controlled, state this explicitly. |  |
| **Blinding** | 5 | Describe who was aware of the group allocation at the different stages of the experiment (during the allocation, the conduct of the experiment, the outcome assessment, and the data analysis). | | Drug administering staff and staff checking animals were blinded. All subsequent analysis was not blinded due to direct comparison of treatment groups being necessary. |
| **Outcome measures** | 6 | a. | Clearly define all outcome measures assessed (e.g. cell death, molecular markers, or behavioural changes). | 1. Spleen size and weight, red blood cell count, HCT, Hb, body weight 2. Not applicable. |
|  |  | b. | For hypothesis-testing studies, specify the primary outcome measure, i.e. the outcome measure that was used to determine the sample size. |  |
| **Statistical**  **methods** | 7 | a. | Provide details of the statistical methods used for each analysis, including software used. | 1. Prism9, paired two-sided T-test. |
|  |  | b. | Describe any methods used to assess whether the data met the assumptions of the statistical approach, and what was done if the assumptions were not met. | 1. Not applicable. |
| **Experimental animals** | 8 | a. | Provide species-appropriate details of the animals used, including species, strain and substrain, sex, age or developmental stage, and, if relevant, weight. | 1. C57BL/6 Jak2V617F homozygous females aged 8-10 weeks. 2. Not applicable |
|  |  | b. | Provide further relevant information on the provenance of animals, health/immune status, genetic modification status, genotype, and any previous procedures. |  |
| **Experimental procedures** | 9 | For each experimental group, including controls, describe the procedures in enough detail to allow others to replicate them, including:   1. What was done, how it was done and what was used. 2. When and how often. 3. Where (including detail of any acclimatisation periods). 4. Why (provide rationale for procedures). | | Methods part:   1. Photographs of spleen, body and spleen weight recorded, Idexx blood analysis, FACS. 2. Body weight was taken once weekly, all other data were obtained at time of sampling. 3. Not applicable 4. Common haematological factors to be assessed in this mice model |
|  |  |  | |  |
| **Results** | 10 | For each experiment conducted, including independent replications, report:   1. Summary/descriptive statistics for each experimental group, with a measure of variability where applicable (e.g. mean and SD, or median and range). 2. If applicable, the effect size with a confidence interval. | | a. average haematocrit of 89.8% ±2.6 (mean±SEM n=4) and HB 445 levels of 22.7g/dl ±2.4 (mean±SEM n=4). reticulocyte counts 9.8x105 446 /ml ±0.73 (mean±SEM n=4) 447 and splenomegaly 198mg±19 (mean±SEM n=4). CBL0137 treatment significantly (p=0.036) reduced the 449 splenomegaly (Fig 8C-D) and reticulocyte (p=0.0005) count  b. Not applicable |
| The Recommended Set | | | | |
| These items complement the Essential 10 and add important context to the study. Reporting the items in both sets represents best practice. | | | | |

**Section/line**

**Item Recommendation number, or reason for not reporting**

| **Abstract** | 11 | Provide an accurate summary of the research objectives, animal species, strain and sex, key methods, principal findings, and study conclusions. | The aim of the research is to investigate the effects of curaxin treatment on MPN hematopoietic stem cells in C57/Bl6 JAK2V617F homozygous female mice by flow cytometry.  Curaxin treatment significantly reduced splenomegaly and reticulocyte counts but had no effect on the stem cells. |
| --- | --- | --- | --- |
| **Background** | 12 | 1. Include sufficient scientific background to understand the rationale and context for the study, and explain the experimental approach. 2. Explain how the animal species and model used address the scientific objectives and, where appropriate, the relevance to human biology. | 1. According to our own in vitro data, curaxin reduces stem cell numbers in vitro. Therefore testing in vivo was the next step. 2. This animal model faithfully recapitulates aspects of Jak2V617F driven MPN. |
| **Objectives** | 13 | Clearly describe the research question, research objectives and, where appropriate, specific hypotheses being tested. | Does curaxin reduce Jak2V617F mutated stem cells in vivo? |
| **Ethical statement** | 14 | Provide the name of the ethical review committee or equivalent that has approved the use of animals in this study, and any relevant licence or protocol numbers (if applicable). If ethical approval was not sought or granted, provide a justification. | All experiments were covered under the UK Home office project licence (licence PD6C67A47; protocols 2 and 5). All experiments were subject to review by the with Animal Welfare and Ethical Review Board (AWERB) of the University of Glasgow. |
| **Housing and husbandry** | 15 | Provide details of housing and husbandry conditions, including any environmental enrichment. | Mice were housed in conventional cages within a licenced, pathogen-free facility, under a 12hr light-dark cycle, at stable temperature (19-23oC) and humidity (55±10%) with ad libitum access to food and water. |
| **Animal care and monitoring** | 16 | 1. Describe any interventions or steps taken in the experimental protocols to reduce pain, suffering and distress. 2. Report any expected or unexpected adverse events. 3. Describe the humane endpoints established for the study, the signs that were monitored and the frequency of monitoring. If the study did not have humane endpoints, state this. | 1. We monitor the mice carefully using body weight and scoring for distress and reduce the drug concentration for the treatment after 2 cycles of curaxin. 2. One mouse died from necrosis. 3. Mice were monitored daily and weighed weekly. Mice were euthanised by CO2 inhalation in a CO2 chamber, cervically dislocated after completion of a 4 week cycle of curaxin treatment. Humane endpoint was established as 10% loss of body weight or a health score of ≥10 or a body conditioning score of 1 or 5. However, this was not reached at any point of the study. |
| **Interpretation/ scientific implications** | 17 | 1. Interpret the results, taking into account the study objectives and hypotheses, current theory and other relevant studies in the literature. 2. Comment on the study limitations including potential sources of bias, limitations of the animal model, and imprecision associated with the results. | 1. Some haematological parameters of Jak2V617F driven MPN can be ameliorated by curaxin treatment. However, stem cell numbers were not reduced with the given sample size. 2. Sample size needs to be increased to draw firm conclusions on the effect of curaxin on the stem cell compartment. |
| **Generalisability/ translation** | 18 | Comment on whether, and how, the findings of this study are likely to generalise to other species or experimental conditions, including any relevance to human biology (where appropriate). | This mouse model faithfully recapitulates some aspects of Jak2V617F driven MPN in humans. Therefore, some relevance to humans is given. |
| **Protocol registration** | 19 | Provide a statement indicating whether a protocol (including the research question, key design features, and analysis plan) was prepared before the study, and if and where this protocol was registered. | No registration of the protocol. Protocol was prepared based on published studies and initial treatment regimen was tested on n=2 WT mice to determine any adverse effects. |
| **Data access** | 20 | Provide a statement describing if and where study data are available. | Study data are available through publication in PLOS One. No other data are available. |
| **Declaration of interests** | 21 | 1. Declare any potential conflicts of interest, including financial and non-financial. If none exist, this should be stated. 2. List all funding sources (including grant identifier) and the role of the funder(s) in the design, analysis and reporting of the study. | 1. Not applicable 2. ADW and AP received awards from Blood Cancer UK (grants 13005 and 19007). TS and FA are supported by Cancer Research UK (grant C5759/A20971 and C5759/A27412. KK was funded by a John Goldman Fellowship sponsored by Leukaemia UK (2019/JGF/003) and CRUK Glasgow Centre funding (C7932/A25142) and CRUK Scotland Centre funding (CTRQQR-2021\100006).   The funders had no role in study design, data collection and analysis, decision to publish, or preparation of the manuscript |


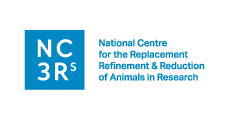
 [www.ARRIVEguidelines.org](http://www.arriveguidelines.org/)
