# Supplementary material for: Identification of curaxin as a potential new therapeutic for JAK2 V617F mutant patients
Source: PLoS One. 2023 May 30;18(5):e0286412. doi: 10.1371/journal.pone.0286412 (PMC10228771; doi:10.1371/journal.pone.0286412)
Supplement: S1 Raw images — (PDF) [file pone.0286412.s003.pdf]

Supplementary Figure S2

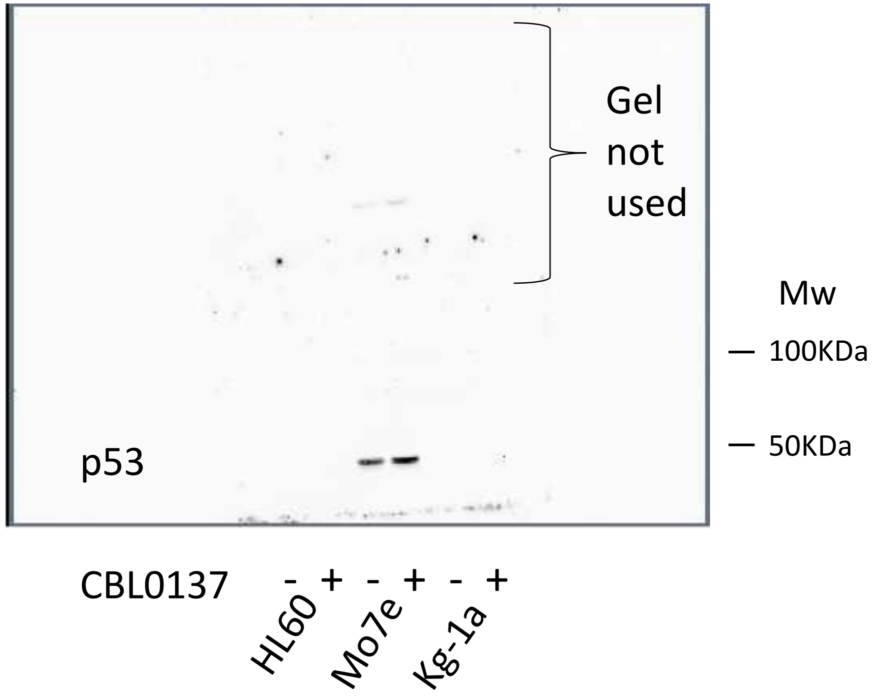

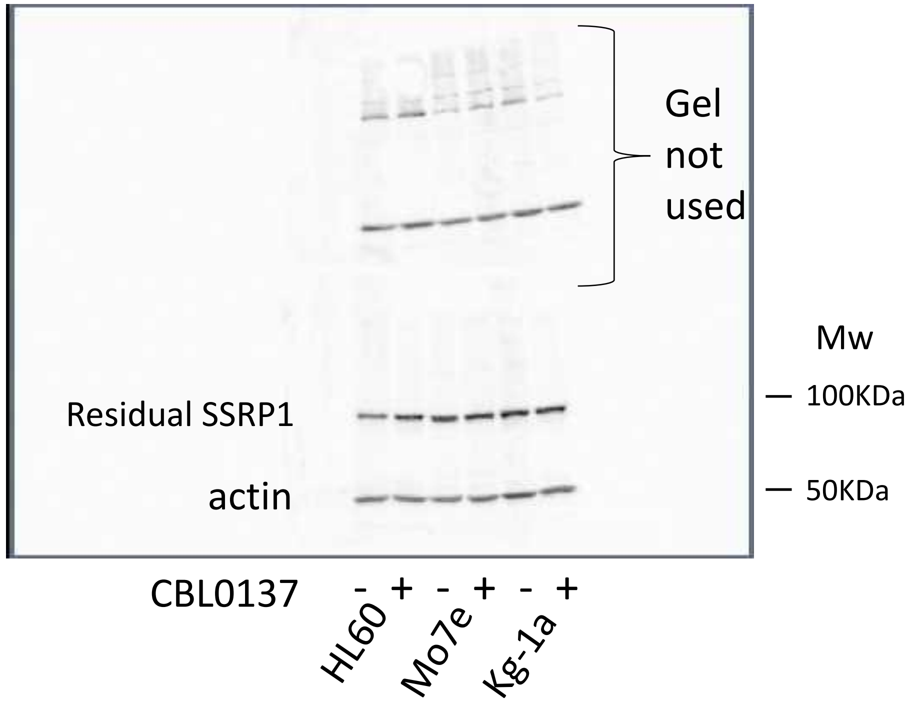

30µg of protein lysate separated on a 7.5% polyacrylamide gel. Probed with anti p53 antibody from NeoMarkers (MS-187-PO) at 1/200 dilution. Anti-mouse IgG HRP conjugated secondary antibody (GE Healthcare NA931) used at 1/5000. Visualised with SuperSignal West Pico chemiluminescent substrate (Thermo Scientific 34578) using BioRad ImageQuant software. Filter was reprobed directly with anti SSRP1 antibody (Cell Signalling 13421) and visualised as in the first stage before reprobing directly with an actin-HRP conjugated antibody (Sigma A3854) at 1/50000

Supplementary Figure S2

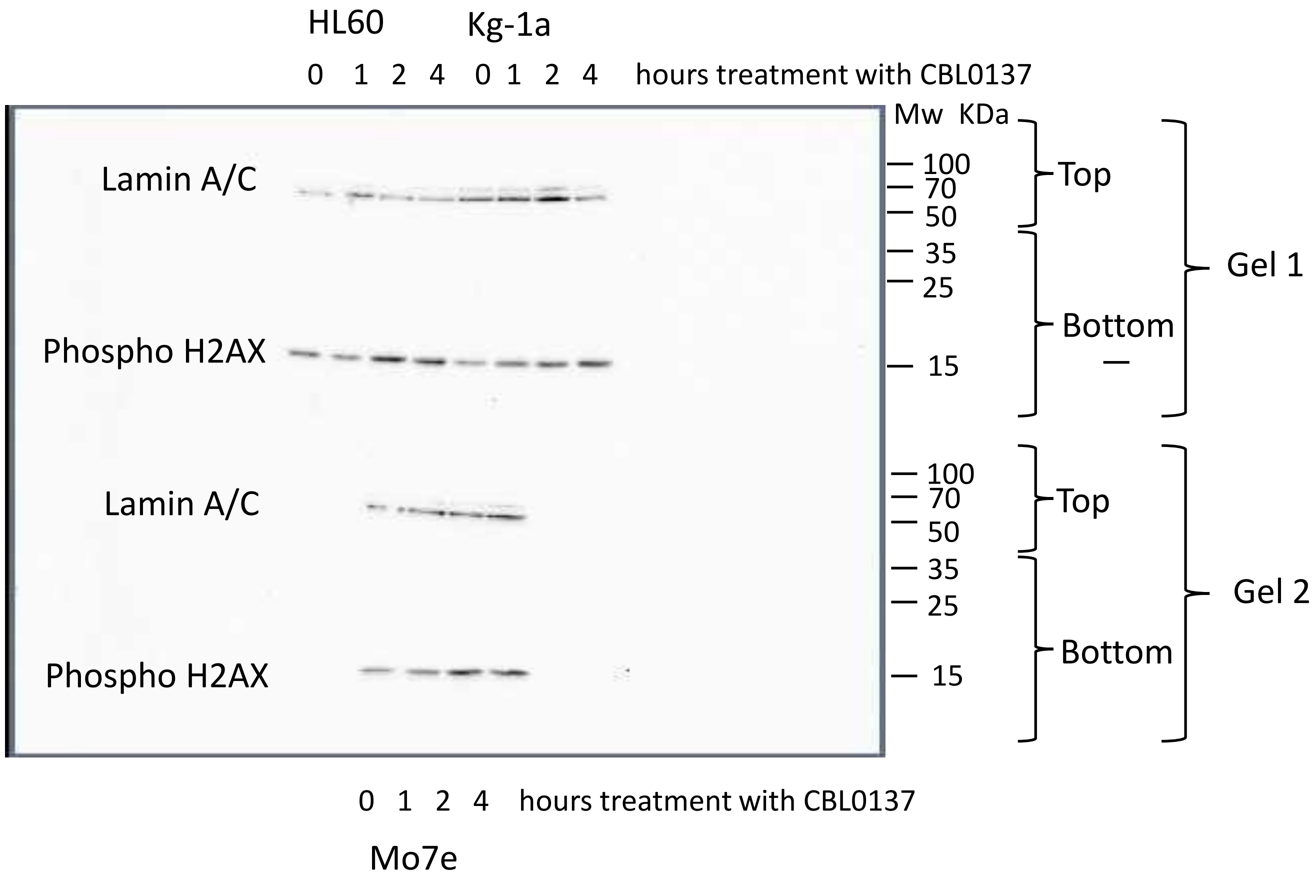

30µg of protein lysate separated on two 12.5% polyacrylamide gels. Following transfer to Nitrocellulose the filters were cut inline with the proteins of around 45KDa. The bottom half was probed with anti phospho H2AX antibody (Cell Signalling #9718) at 1/1000 dilution and the top half with anti Lamin A/C (Cell Signalling #2032) at 1/1000 dilution. Anti-rabbit IgG HRP conjugated secondary antibody (GE Healthcare NA934) used at 1/5000. All four filters were visualised simultaneously with SuperSignal West Pico chemiluminescent substrate (Thermo Scientific 34578) using BioRad ImageQuant software
